# 885. Pregnancy Outcomes and Pharmacokinetics in Pregnant Women Living with HIV Exposed to Long-Acting Cabotegravir and Rilpivirine in Clinical Trials

**DOI:** 10.1093/ofid/ofab466.1080

**Published:** 2021-12-04

**Authors:** Parul Patel, Susan L Ford, Mark Baker, Claudia Meyer, Louise Garside, Ronald D’Amico, Rodica Van Solingen-Ristea, Herta Crauwels, Joseph Polli, Ciara Seal, Shanker Thiagarajah, Eileen Birmingham, William Spreen, Bryan Baugh, Matthew Bosse, Vani Vannappagari

**Affiliations:** 1 ViiV Healthcare, Research Triangle Park, NC; 2 GlaxoSmithKline, Research Triangle Park, NC; 3 Janssen Research & Development, LLC, Beerse, Antwerpen, Belgium; 4 Janssen Research and Development, Antwerpen, Oost-Vlaanderen, Belgium

## Abstract

**Background:**

Limited data exist among women living with HIV who become pregnant while exposed to long-acting (LA) cabotegravir (CAB) and rilpivirine (RPV). We report outcomes in pregnant participants and LA pharmacokinetic (PK) tail data in pregnant women exposed to CAB+RPV with live births.

**Methods:**

Women of reproductive potential exposed to ≥ 1 dose of CAB+RPV (oral/LA) from ViiV-sponsored Phase 2/3/3b clinical treatment studies and the compassionate use program were included in this analysis and pregnancies identified. Per protocol, upon identification of pregnancy, CAB+RPV was discontinued and an alternative regimen initiated, with continued quarterly PK sampling for 52 weeks post last injection during long-term safety follow-up (LTFU). Descriptive characteristics of pregnant women and birth outcomes and available CAB and RPV PK during pregnancy for those with live births are summarized.

**Results:**

As of March 31, 2021, 26/325 women of reproductive potential (age 18–49 years) became pregnant while exposed to CAB+RPV (5 oral, 21 LA [including 3 following LA discontinuation]). There were 11 live births (1 oral, 10 LA), of which 10 had no reported congenital abnormalities and 1 had reported congenital ptosis, in a pre-term infant with intrauterine growth restriction. There were 9 elective terminations and 6 miscarriages (5 in first 9 weeks of gestation). Ten women exposed to intramuscular CAB+RPV LA became pregnant with subsequent live birth outcomes, including 3 infants conceived during the PK tail in LTFU. All women were virologically suppressed at time of pregnancy identification. In women becoming pregnant on LA dosing, plasma CAB and RPV concentrations during pregnancy were within the range of expected concentrations in non-pregnant women. Two of 10 women with live births exposed to CAB+RPV LA continued LA therapy during pregnancy (compassionate use program participants).

**Conclusion:**

Pregnancy outcomes in women exposed to CAB+RPV at conception are consistent with earlier findings. There was 1 reported congenital anomaly among 11 live births. CAB and RPV PK tail in pregnancy was within the expected range for non-pregnant women. Ongoing monitoring of birth defects within the antiretroviral pregnancy registry and pregnancy surveillance within the treatment program continues.

**Disclosures:**

**Parul Patel, PharmD**, **GlaxoSmithKline** (Shareholder)**ViiV Healthcare** (Employee) **Susan L. Ford, PharmD**, **GlaxoSmithKline** (Shareholder)**ViiV Healthcare** (Employee) **Mark Baker, PhD**, **GlaxoSmithKline** (Shareholder)**ViiV Healthcare** (Employee) **Claudia Meyer, MBChB, MRCP, MSc, FRCPath, DTM&H**, **GlaxoSmithKline** (Employee, Shareholder) **Louise Garside, PhD**, **GlaxoSmithKline** (Employee) **Ronald D’Amico, DO, MSc**, **GlaxoSmithKline** (Shareholder)**ViiV Healthcare** (Employee) **Rodica Van Solingen-Ristea, MD**, **Janssen Research and Development** (Employee)**ViiV Healthcare** (Employee) **Herta Crauwels, PhD**, **Janssen** (Employee) **Joseph Polli, PhD, FAAPS**, **GlaxoSmithKline** (Shareholder)**ViiV Healthcare** (Employee) **Ciara Seal, BS**, **GlaxoSmithKline** (Employee, Shareholder) **Shanker Thiagarajah, MB ChB**, **GlaxoSmithKline** (Employee, Shareholder) **Eileen Birmingham, MD, MPH**, **Janssen Research and Development** (Employee, Shareholder) **William Spreen, PharmD**, **GlaxoSmithKline** (Shareholder)**ViiV Healthcare** (Employee) **Bryan Baugh, MD**, **Janssen, Johnson & Johnson** (Employee, Shareholder) **Matthew Bosse, DO**, **GlaxoSmithKline** (Shareholder)**ViiV Healthcare** (Employee) **Vani Vannappagari, MBBS, MPH, PhD**, **ViiV Healthcare Limited** (Employee)

